# 639. A Decade-Long Analysis of Ventilator-Associated Pneumonia in the Intensive Care Unit of a Public Hospital in a Major Brazilian City: An Exploration of Trends, Challenges, and Opportunities

**DOI:** 10.1093/ofid/ofae631.204

**Published:** 2025-01-29

**Authors:** Flávia Eniko Pinto, Adrielle Rodrigues, Gleiciane M Teixeira, Samara Mariana Da Silva, Simony Gonçalves, Bráulio R G M Couto

**Affiliations:** Hospital Risoleta Tolentino Neves - HRTN, Belo Horizonte, Minas Gerais, Brazil; Hospital Risoleta Tolentino Neves - HRTN, Belo Horizonte, Minas Gerais, Brazil; Hospital Risoleta Tolentino Neves - HRTN, Belo Horizonte, Minas Gerais, Brazil; Hospital Risoleta Tolentino Neves - HRTN, Belo Horizonte, Minas Gerais, Brazil; Hospital Risoleta Tolentino Neves - HRTN, Belo Horizonte, Minas Gerais, Brazil; AMECI – Associação Mineira de Epidemiologia e Controle de Infecções, Belo Horizonte, Minas Gerais, Brazil

## Abstract

**Background:**

Ventilator-associated pneumonia (VAP) remains a critical issue in ICUs. This study investigates VAP dynamics over the past decade by examining trends in its incidence, shifts in etiological agents and their antimicrobial sensitivities, and factors affecting patient mortality.

**Figure 1:**
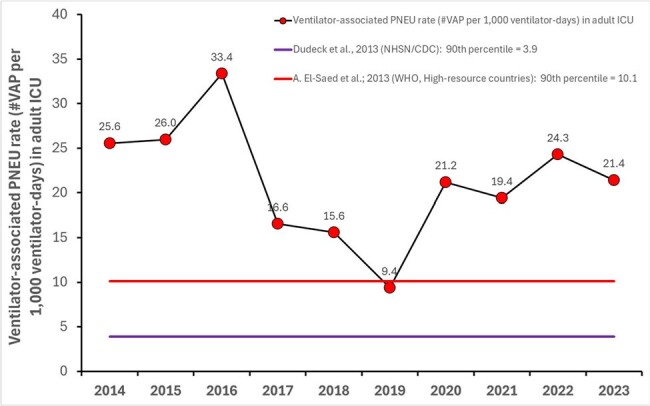
Ten-Year Trend of Ventilator-Associated Pneumonia Incidence in the ICU of a Major Brazilian Public Hospital: Initial Reduction Reversed in 2020 Due to the COVID-19 Pandemic; Current Levels (2021-2023) Show a 23% Decrease Compared to the Start of the Series (2014-2016).

Ten-Year Trend of Ventilator-Associated Pneumonia Incidence in the ICU of a Major Brazilian Public Hospital: Initial Reduction Reversed in 2020 Due to the COVID-19 Pandemic; Current Levels (2021-2023) Show a 23% Decrease Compared to the Start of the Series (2014-2016).

**Methods:**

Surveillance of ICU patients on mechanical ventilation in a 300-bed public hospital in Belo Horizonte, Brazil (Jan/2014-Dec/2023) followed the National Healthcare Safety Network protocol. Logistic regression analyzed VAP mortality risks. Microorganism clusters were identified using dynamic programming to determine edit distances between pseudoDNA strings, which were formed from concatenated antimicrobial sensitivity results (S, R, I) for each strain.

**Figure 2:**
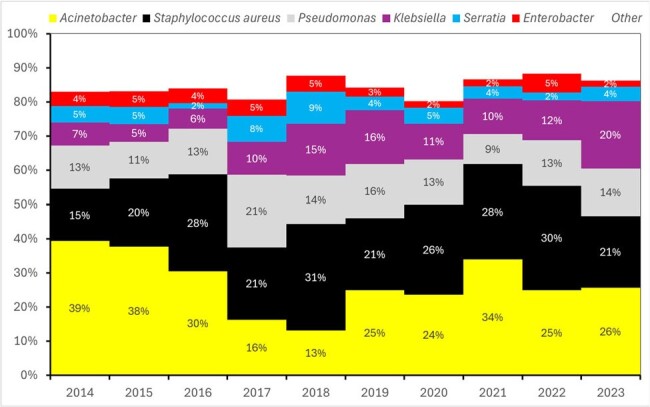
Ten-Year Trends in VAP Etiology in the ICU of a Major Brazilian Public Hospital: Despite Variations in Predominant Agents, the Same Bacteria (e.g., Acinetobacter, Staphylococcus aureus, Pseudomonas, Klebsiella, Serratia) Have Consistently Caused VAP Throughout the Decade.

Ten-Year Trends in VAP Etiology in the ICU of a Major Brazilian Public Hospital: Despite Variations in Predominant Agents, the Same Bacteria (e.g., Acinetobacter, Staphylococcus aureus, Pseudomonas, Klebsiella, Serratia) Have Consistently Caused VAP Throughout the Decade.

**Results:**

From 2014 to 2023, there were 1,166 VAP cases (20.9 cases per 1,000 ventilator-days). Initially, the incidence of VAP decreased significantly until 2019, but a rising trend began in 2020 and persisted through 2022 (Fig. 1). The predominant etiological agents, Acinetobacter, Staphylococcus aureus, Pseudomonas, and Klebsiella, accounted for 70%-80% of cases (Fig. 2). Antimicrobial sensitivity remained relatively stable over the decade, with a notable decline only in 2023 (Fig. 3). PseudoDNA analysis for Acinetobacter baumannii revealed two clusters: a small multisensible cluster (4% of strains) and a larger multiresistant cluster present throughout the decade (Fig. 4). The same pattern was observed for the other predominant microorganisms. A logistic regression model predicting mortality in VAP patients identified four significant predictors (area under the ROC curve = 0.68): patient age, time from hospitalization to pneumonia diagnosis, secondary sepsis (Odds Ratio = 2.6), and number of prior hospitalizations at the same hospital.

**Figure 3:**
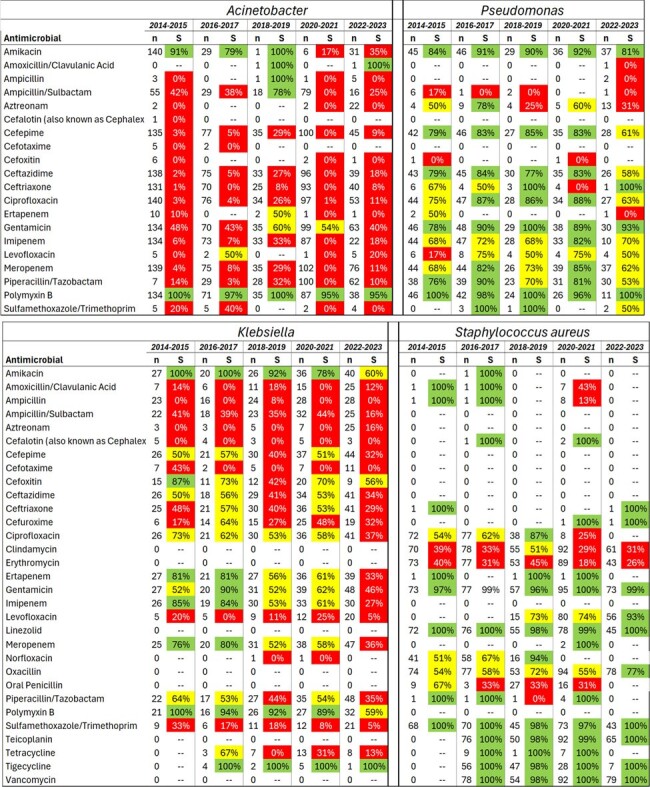
Ten-Year Trends in Antimicrobial Sensitivity of Acinetobacter, Pseudomonas, Klebsiella and Staphylococcus aureus Causing VAP in the ICU of a Major Brazilian Public Hospital: Increased Resistance Observed in 2023.

Ten-Year Trends in Antimicrobial Sensitivity of Acinetobacter, Pseudomonas, Klebsiella and Staphylococcus aureus Causing VAP in the ICU of a Major Brazilian Public Hospital: Increased Resistance Observed in 2023.

**Conclusion:**

We addressed three key questions on VAP: 1) Despite a temporary increase in 2020, we achieved a 23% overall reduction in VAP incidence, though it remains above benchmark levels. 2) The etiology of VAP has been consistent over the decade, but antimicrobial sensitivity has deteriorated. 3) Age, time from hospitalization to pneumonia diagnosis, prior admissions, and notably, secondary sepsis, are significant risk factors for VAP mortality.

**Figure 4:**
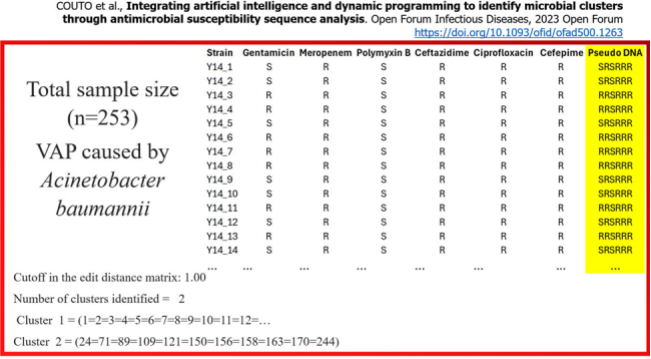
PseudoDNA Cluster Analysis of Acinetobacter baumannii Showing Two Distinct Groups: A Small Multisensible Cluster (4% of Strains) and a Predominant Multiresistant Cluster Persistent Over the Decade

PseudoDNA Cluster Analysis of Acinetobacter baumannii Showing Two Distinct Groups: A Small Multisensible Cluster (4% of Strains) and a Predominant Multiresistant Cluster Persistent Over the Decade

**Disclosures:**

**All Authors**: No reported disclosures

